# Tuberculosis in Healthcare Workers and Infection Control Measures at Primary Healthcare Facilities in South Africa

**DOI:** 10.1371/journal.pone.0076272

**Published:** 2013-10-02

**Authors:** Mareli M. Claassens, Cari van Schalkwyk, Elizabeth du Toit, Eline Roest, Carl J. Lombard, Donald A. Enarson, Nulda Beyers, Martien W. Borgdorff

**Affiliations:** 1 Desmond Tutu Tuberculosis Centre, Department of Paediatrics and Child Health, Stellenbosch University, Parow, South Africa; 2 The South African Department of Science and Technology / National Research Foundation Centre of Excellence in Epidemiological Modelling and Analysis, Stellenbosch University, Stellenbosch, South Africa; 3 Biostatistics Unit, Medical Research Council, Parow, South Africa; 4 The International Union Against Tuberculosis and Lung Disease, Paris, France; 5 Department of Clinical Epidemiology, Biostatistics and Bio-informatics, University of Amsterdam, Amsterdam, The Netherlands; McGill University, Canada

## Abstract

**Background:**

Challenges exist regarding TB infection control and TB in hospital-based healthcare workers in South Africa. However, few studies report on TB in non-hospital based healthcare workers such as primary or community healthcare workers. Our objectives were to investigate the implementation of TB infection control measures at primary healthcare facilities, the smear positive TB incidence rate amongst primary healthcare workers and the association between TB infection control measures and all types of TB in healthcare workers.

**Methods:**

One hundred and thirty three primary healthcare facilities were visited in five provinces of South Africa in 2009. At each facility, a TB infection control audit and facility questionnaire were completed. The number of healthcare workers who had had TB during the past three years was obtained.

**Results:**

The standardised incidence ratio of smear positive TB in primary healthcare workers indicated an incidence rate of more than double that of the general population. In a univariable logistic regression, the infection control audit score was significantly associated with reported cases of TB in healthcare workers (OR=1.04, 95%CI 1.01-1.08, p=0.02) as was the number of staff (OR=3.78, 95%CI 1.77-8.08). In the multivariable analysis, the number of staff remained significantly associated with TB in healthcare workers (OR=3.33, 95%CI 1.37-8.08).

**Conclusion:**

The high rate of TB in healthcare workers suggests a substantial nosocomial transmission risk, but the infection control audit tool which was used did not perform adequately as a measure of this risk. Infection control measures should be monitored by validated tools developed and tested locally. Different strategies, such as routine surveillance systems, could be used to evaluate the burden of TB in healthcare workers in order to calculate TB incidence, monitor trends and implement interventions to decrease occupational TB.

## Introduction

The World Health Organization (WHO) and Centers for Disease Control and Prevention (CDC) have proposed practical low cost interventions to reduce nosocomial transmission of *Mycobacterium tuberculosis* in resource limited settings [1,2]. TB disease in healthcare workers can be used as a proxy to quantify nosocomial TB transmission in low and middle income countries such as Thailand and Malawi [3]. Evidence from systematic reviews reinforces the need to design and implement simple, effective and affordable TB infection control measures in healthcare facilities [3,4,5]. Such measures conserve resources in terms of direct and indirect costs and reduce the TB burden [1]. To evaluate the effectiveness of infection control measures, the CDC has developed a TB infection control audit tool [2] aiming to be applicable to different settings.

Studies have been published internationally and in South Africa about TB in healthcare workers. Five per cent of healthcare workers in a study from Uganda reported having had TB in the past five years [6]. In Nigeria 3.3% of healthcare workers were acid fast bacilli positive [7]. A study from India showed healthcare workers employed in medical wards who had frequent contact with any patients had a higher odds of developing TB [8]. A case series from KwaZulu-Natal in South Africa [9] reported the psychosocial impact of drug resistant TB on five human immunodeficiency virus (HIV) negative doctors who, after they recovered from their illness and because of their disease experience, had minimal or no involvement with TB patients. Another study [10] reported four of the ten extremely drug resistant TB cases had died by the time of publication. O’Donnell et al [11] reported an incidence rate ratio of 5.5 for multidrug-resistant (MDR) TB hospital admissions in healthcare workers compared to the general population. A tertiary hospital reported both drug sensitive and drug resistant TB were potentially transmitted nosocomially [12,13]. Other studies reported poor infection control measures at primary healthcare facilities [14] and TB hospitals admitting drug resistant cases [15].

Substantial challenges thus exist regarding TB infection control and TB in hospital-based healthcare workers in South Africa. However, few studies report on TB in non-hospital based healthcare workers such as primary or community healthcare workers. A standardised TB incidence ratio of 2.5 was shown amongst community-based healthcare researchers in comparison to the communities where they worked and lived [16] and a TB prevalence of 5% was documented amongst community healthcare workers in Cape Town [17] albeit in a small non-representative sample. TB in primary healthcare workers has not yet been described in the South African context.

The objectives of this study were to investigate the implementation of TB infection control measures at primary healthcare facilities in five provinces of South Africa, the smear positive TB incidence rate in healthcare workers and the association between TB infection control measures and all types of TB in healthcare workers. 

## Methodology

### Ethics

Ethics approval was obtained from Stellenbosch University (N09/02/038) and the Ethics Advisory Group of the International Union against Tuberculosis and Lung Disease (03/2009). Questionnaires were barcoded for confidentiality and quality control. Facility names were deleted from the database and anonymously linked by the data manager. Facility managers signed informed consent prior to enrollment. Permission to do the study in the provinces was obtained via the National Department of Health.

### Study design

In a cross sectional ecological study 133 primary healthcare facilities were visited between May and September 2009 in five provinces of South Africa. The unit of investigation was a primary healthcare facility. The facilities were systematically sampled from a list of facilities supported by the University Research Corporation (URC) as part of their Technical Assistance and Support Contract II, Tuberculosis (TASC II TB) project [18] in districts identified by the National TB Crisis Plan as areas performing poorly with regards to the TB Programme and comprising 20% of the TB burden in South Africa. The TASC II TB project reached 659 facilities in 11 districts over a period of five years.

### Definition

A healthcare worker was defined as any individual employed at the facility.

### Exposures and outcome

At each facility, the research team completed a TB infection control audit and a questionnaire answered by the facility manager or another focal person, who was defined as a healthcare worker working in the TB room with knowledge of the TB programme.

The main determinant, TB infection control measures, was evaluated by using an audit tool at a single point in time at each facility. This tool included questions about administrative, environmental and personal respiratory protection measures as specified in the CDC template [2]. A score was calculated by counting the number of ‘yes’ responses (indicating a good infection control measure) which was scored 1, ‘no’ responses (indicating a poor infection control measure) which was scored -1 and ‘unknown’ or ‘not applicable’ responses which was scored 0. The total score was treated as a continuous variable with a higher score indicating better infection control. Items were not weighted. The research team received training on how to perform an infection control audit before the study commenced.

Information on smear positive TB and other types of TB amongst healthcare workers was captured in the questionnaire. The total number of healthcare workers who had had TB at each facility during the period January 2006 through December 2008 was obtained from the questionnaire. Individual data of healthcare workers were not captured. TB infection status or HIV data were not included in the study. The questionnaire included questions on the number of staff at each facility by calendar year, the geographical location of the facility (rural/urban and province), whether the facility had a fast track for TB suspects or patients and/or an area designated specifically for the treatment of TB patients, whether the facility had an occupational health policy and whether healthcare workers were screened for TB.

### Sample size calculation

The hypothesis was that the Pearson correlation coefficient between the TB incidence rate in healthcare workers and the audit tool score would be ρ=0.3, with the assumption that a linear relationship exists. A 0.05 two-sided Fisher’s z-test had 90% power for ρ=0.3 to be significant when the sample size was 113; 30 facilities were systematically sampled from a random starting point on the URC list for each province except for province 5 where only 16 facilities were supported by URC (where all were selected). A total of 136 facilities were selected to account for the possibility of missing data. Of these facilities, 133 were visited of which 12 were excluded from the analyses as they were unable to provide any information about TB in healthcare workers. Three facilities in Mpumalanga province were not visited because of time constraints.

### Data analysis

The smear positive TB incidence rate in healthcare workers was calculated as the number of healthcare workers who developed smear positive TB (numerator) divided by the total number of healthcare workers (denominator) for each of the three years included in the study. In order to compare the TB incidence rate among healthcare workers with the general population the incidence rate was combined for all facilities. For comparison with the general population we calculated a standardised incidence ratio, using indirect standardisation for age and sex, as was done in other studies [19,20].

**Figure 1:**
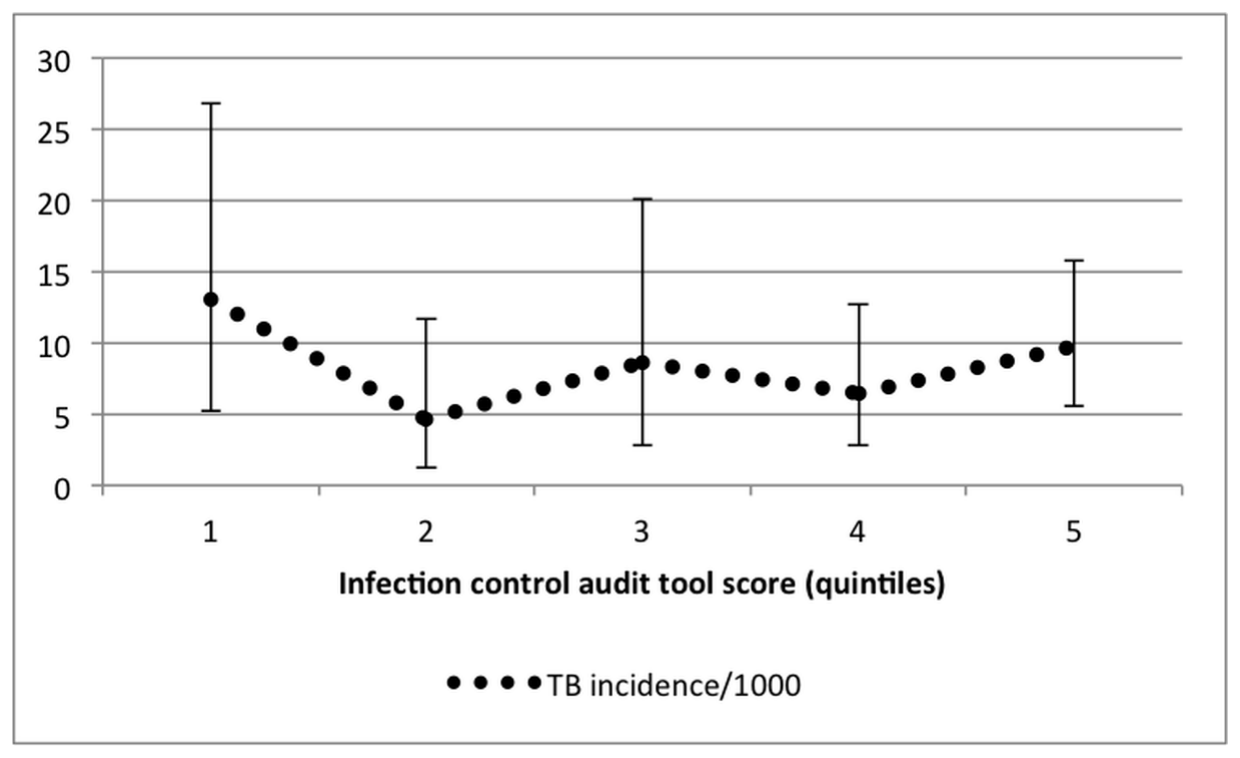
Smear positive TB incidence^α^ according to infection control audit tool score quintiles for 2008*. ^α^Smear positive TB incidence did not differ significantly between quintiles. *A higher quintile is an indication of better infection control measures.

As 64% of the facilities did not report any healthcare workers with TB (all types) for the study period, we then classified the facilities according to whether or not there was any healthcare worker with TB in the study period. This binary variable was used as the primary outcome in a logistic regression analysis. The audit tool score (as a continuous variable) was investigated as primary determinant. The association between the score and smear positive TB incidence for 2008 is graphically demonstrated by categorising the score into quintiles (Figure 1). The variable for the number of staff per facility did not have a normal distribution and was logistically transformed (natural logarithm) before inclusion as a possible confounder. For the multiple imputation of missing values, the imputation model included all the variables investigated as predictors of TB in healthcare workers. Data were analysed using STATA 12 (StataCorp LP, College Station, TX, USA).

## Results

### TB incidence rate

The smear positive TB incidence rate per 100,000 persons was 834 (95%CI 431-1457) in 2006, 1,092 (95%CI 647-1725) in 2007 and 887 (95%CI 517-1420) in 2008 across the five provinces for all facilities (including those where there were no TB cases in healthcare workers). The standardised incidence ratio (Table 1) was 2.4 (95%CI 1.2-4.2) in 2006, 3.0 (95%CI 1.8-4.7) in 2007 and 2.3 (95%CI 1.3-3.7) in 2008.

**Table 1:** Standardised incidence ratios (SIR) for smear positive TB in healthcare workers (January 2006-December 2008).

Year observed	Number of staff in study	Observed cases	Expected cases	SIR	Chi-square	P-value	95% CI	
2006	1439	12	5.04	2.38	9.62	0.002	1.23	4.16
2007	1649	18	6.04	2.98	23.65	<0.001	1.77	4.71
2008	1917	17	7.41	2.29	12.41	<0.001	1.34	3.67

### Infection control

The range of the total score of the audit tool was -22 to +51 (Table 2). The mean number of staff per facility was 17 (IQR 8-23) at the visit. Forty four facilities (36%) had had healthcare workers with TB disease in the study period. Sixty six facilities (55%) were urban. Facilities were distributed across provinces with the most facilities from province 1 (n=28) and fewest from province 5 (n=14). Seventy seven facilities (64%) had a TB area/room and 106 (88%) a fast track for TB suspects/patients. Forty eight facilities (40%) had an occupational health policy and 43 (36%) reported TB screening for healthcare workers.

**Table 2:** Descriptive characteristics of facilities included in the analysis (n=121).

	*Range*	Mean (SD)*
Infection control audit tool score		
*total*	(-22;51)	9 (12)
*administrative*	(-4;19)	8 (4)
*environmental*	(-9;16)	6 (4)
*personal*	(-9;8)	-3 (3)
Number of staff (25 missing)	(3;124)	17 (15)
	*Number*	*%*
Facility with TB in HCW		
*yes*	44	36
*no*	77	64
Geographical location		
*urban*	66	55
*rural*	55	45
Province		
*1*	28	23
*2*	24	20
*3*	26	21
*4*	29	24
*5*	14	12
TB area/room at facility		
*yes*	77	64
*no*	44	36
Fast track for TB patients (1 missing)		
*yes*	106	88
*no*	14	12
Occupational health policy (26 unknown)		
*yes*	48	51
*no*	47	49
TB screening for HCW (1 missing)		
*yes*	43	36
*no*	77	64

* SD = standard deviation

Administrative control measures were assessed with the audit tool (Table 3). The major differences between facilities were: 28 of 44 facilities with TB cases (64%) separated infectious patients from non-infectious patients compared to 32 of 77 facilities (42%) without TB cases. Forty facilities (91%) with TB cases trained healthcare workers on infection control compared to 50 facilities (65%) without TB cases. With regards to environmental controls, 33 facilities (75%) with TB cases reported the use of cross ventilation compared to 52 facilities (68%) without TB cases. 23 (52%) facilities with TB cases used propeller fans compared to 56 (73%) facilities without TB cases. Thirty facilities (68%) with TB cases reported exhaust ventilation systems compared to forty two facilities (55%) without TB cases. Eight facilities (18%) with TB cases had previously had audits compared to one facility (1%) without TB cases. Overall only four facilities (3%) had an area designed to separate possible or confirmed MDR-TB cases and six facilities (5%) had a written respiratory protection plan.

**Table 3:** Distribution of infection control measures among facilities with/out TB in healthcare workers.

	With TB (44)		Without TB (77)	
	Yes*	%	Yes	%
**Administrative controls**				
Is there someone in charge of TB infection control at the healthcare facility?	21	48	34	44
Is there a written TB infection control plan in place?	9	20	14	18
Does the infection control plan or standard clinic procedures, if no specific infection control plan is in place, allow for:				
*Early detection of TB as evidenced by time between taking of sputum and receiving of results? (i.e. within 48 hours)*	29	66	55	71
*Early treatment of infectious TB patients? (i.e. treatment started within 5 days of receiving positive sputum results)?*	37	84	65	84
*Separation of Infectious patients?*	28	64	32	42
*With reference to MDR-TB patients: Are patients separated in the clinic whilst they are awaiting transport to a MDR-TB treatment facility?*	17	39	35	45
Are healthcare workers trained on TB infection control practices?	40	91	50	65
Are patients and/or their families educated on TB infection control practices?	44	100	77	100
**Environmental controls**				
What environmental controls are used in the healthcare facility?				
*Natural Ventilation*	44	100	75	97
*Open Windows Policy*	35	80	62	81
*Cross Ventilation*	33	75	52	68
*Propeller Fans*	23	52	56	73
*Exhaust Ventilation Systems*	30	68	42	55
*HEPA Filters*	0	0	1	1
*UVGI Lights*	13	30	5	6
Are there any areas designed to separate MDR-TB suspected or confirmed cases?	2	5	2	3
Does the facility have access to an engineer or other professional for assistance on design, installation, maintenance and assessment of environmental controls?	41	93	73	95
Are environmental controls periodically maintained with results written down in registers?	16	36	19	25
**Personal respiratory protection**				
Is there a written respiratory protection plan in the healthcare facility?	1	2	5	6
Are there N95 respirators available for staff to use?	13	30	24	31
Are staff trained on respiratory protection?	9	20	11	14
**Previous infection control audits**				
Has a TB infection control audit been performed at the healthcare facility?	8	18	1	1

* if not 'yes', participants could answer 'no' or 'unknown' which were grouped for this table

### Infection control measures as risk factors for TB

The smear positive TB incidence for 2008 per audit tool score quintile is shown in Figure 1, indicating a similar TB incidence in all quintiles. In the univariable logistic regression (Table 4), the total audit tool score as a continuous variable was significantly associated (per unit of the score) with whether the facility had TB (all types) in healthcare workers (OR=1.04, 95%CI 1.01-1.08, p=0.02) implying that facilities with TB cases had better infection control measures. Significant associations were observed for environmental controls (OR=1.12, 95%CI 1.01-1.23), number of staff at a facility (OR=3.78, 95%CI 1.77-8.08) which means a 3.78 increase in odds for every 2.72 (natural logarithm) increase in the number of staff, and whether a facility had a TB room/area (OR=3.24, 95%CI 1.37-7.65).

**Table 4:** Logistic regression models with TB (all types) in healthcare workers as binary outcome (n=121 facilities).

		Univariable				Multivariable^α^			
		OR	95%CI		P-value	OR	95%CI		P-value
Audit tool score*									
	Total	1.04	1.01	1.08	0.02	1.01	0.97	1.06	0.54
	Administrative	1.09	0.99	1.19	0.07				
	Environmental	1.12	1.01	1.23	0.03				
	Personal	1.04	0.91	1.20	0.54				
Number of staff (log-scale)		3.78	1.77	8.08	<0.01	3.33	1.37	8.08	0.01
Geographical location									
	Urban	1.00							
	Rural	1.33	0.63	2.80	0.45				
Province									
	1	1.00		overall p=	0.01			overall p=	0.03
	2	0.66	0.18	2.37	0.52	0.86	0.20	3.79	0.84
	3	1.83	0.59	5.68	0.29	0.87	0.23	3.25	0.84
	4	0.95	0.30	3.02	0.93	0.91	0.22	3.77	0.90
	5	15.00	2.72	82.67	<0.01	11.15	1.84	67.39	0.01
TB area/room at facility									
	no	1.00							
	yes	3.24	1.37	7.65	0.01	1.67	0.52	5.43	0.39
Fast track for TB patients									
	no	1.00							
	yes	2.29	0.60	8.72	0.22				
Occupational health policy									
	no	1.00							
	yes	1.61	0.73	3.53	0.24				
TB screening for HCW									
	no	1.00							
	yes	1.92	0.89	4.15	0.10				

* when the components of the audit tool were modelled separately in the multivariable analysis, none were significantly associated with the outcome or each other.

α Only the variables significantly associated with the outcome were included in the multivariable analysis.

Although facilities with more staff had a higher score (coeff 5.69, 95%CI 2.24-9.14, p=0.002), there was no interaction between the number of staff and the score in association with whether the facility had any TB cases (data not shown). The separate scores for administrative and personal controls, geographic location and whether a facility had a fast track for TB patients, an occupational health policy or health screening for staff were not associated with TB in healthcare workers. In the multivariable analysis, the number of staff (OR=3.33, 95%CI 1.37-8.08) remained significantly associated with TB in healthcare workers indicating a confounding effect.

## Discussion

In our study the standardised incidence ratio for smear positive TB in primary healthcare workers indicated an incidence rate more than double that of the general population for each of the three years. TB in healthcare workers (all types of TB) had a weak association with infection control measures in the unadjusted models, particularly with environmental measures, indicating the relative importance of these measures and mirroring findings showing the effectiveness of natural ventilation [21]. Our results indicate that (i) occupational TB is concerning amongst primary healthcare workers and (ii) the audit tool did not perform as expected as a measure of nosocomial transmission risk since we found inverse associations, in other words the presence of nosocomial transmission risk was associated with better infection control measures.

TB incidence in healthcare workers in South Africa was previously (1986-1997) reported [22] as significantly lower than in the general population [23], but in hospital-based healthcare workers it increased from 1,024 to 1,641 per 100,000 (1999-2003), significantly higher than in the general population [24]. In our study, we focused on healthcare workers at primary healthcare facilities, a cadre not previously investigated in South Africa. We showed an occupational risk of TB similar to the risk in hospital-based healthcare workers indicating an urgent need for interventions to limit occupational exposure. However, because of the high TB incidence in the general population in South Africa, ways of measuring occupational risk *per se* should be investigated.

Our study also focused on TB infection control. Infection control audits are characterised by a cycle of four parts [25,26]: (i) standards are set, (ii) infection control measures and outcomes are evaluated against these standards, (iii) measures are corrected if needed and (iv) re-auditing completes the cycle. In general, nosocomial infection rates are the outcome of primary interest for infection control audits [26], albeit the most difficult to evaluate. Repeated surveys could be used to measure and compare infection rates in healthcare workers [26,27]. Our study evaluated TB infection control measures taking into account that the South African Department of Health developed TB infection control and occupational TB guidelines in 2007 [28]. According to an infection control audit cycle, we were evaluating the measures and outcomes against the standards. However, data on TB in healthcare workers were not routinely captured and we depended on information gathered from a focal person at each facility. This is in contrast to other areas, for instance a routine electronic notification system used in Samara Oblast (Russia) capturing individual demographic, clinical and epidemiological data, including employment information [29] to calculate TB incidence in healthcare workers.

Of interest is the possible association between infection control measures and TB in healthcare workers. We could not identify studies where this association was investigated despite numerous studies on TB infection control [3,4,5,14,30] as proposed by the Stop TB 3 I’s strategy [31,32]. However, after adjusting for the number of staff per facility no association between the audit score and whether a facility had had healthcare workers with TB was found. These findings lead to speculation as to whether the tool was a sufficient indicator of infection control.

Firstly, the question should be asked whether the same audit tool could be used in different contexts. In countries with a low TB incidence like the United States of America (USA), the implementation of infection control guidelines has proven to be effective in the prevention of outbreaks of nosocomial disease and in decreasing the rate of infection in healthcare workers [2] but a recent study comparing China with the USA [33] indicated that an infection control manual developed by the China Centers for Disease Control did not include data from China and was based mostly on expert opinion. Ideally countries should study local infection control measures to inform their guidelines and tools, for instance studies to identify problems, evaluate new policies and monitor the implementation of policies [33], rather than using tools developed for different health systems, disease burdens and resources. For instance, a tool derived from the CDC template but focusing on specific elements (developed by the Wits Reproductive Health and HIV institute) [34] could be evaluated in the South African context. Secondly, the rationale for doing infection control audits should be re-evaluated. If audits do not give an indication of disease in healthcare workers as a proxy for nosocomial transmission but are used only as a process management tool when healthcare workers are overburdened already, is it worthwhile?

Infection control is a cardinal part of healthcare facilities, whether hospitals or primary facilities. However, when evaluating the effectiveness of infection control measures the measurement tools should be validated before programmatic implementation to ensure that a false sense of security amongst healthcare workers does not mask nosocomial transmission.

### Limitations

Our study was limited by the measurement of TB in healthcare workers over a period of three years, while the infection control audit was done at a single point in time. Facilities may have changed their practices prior to the audit, for instance the facilities with the highest risk may have implemented infection control measures and these changes would not be reflected in our study, thereby introducing the possibility of reverse causality. Future investigators should consider prospective cohorts of healthcare workers with regular infection control audits and routine surveillance in health facilities.

We depended on information from a single source about TB in healthcare workers. This may have underestimated the number of healthcare workers with TB, but it also meant HIV status could not be captured. However, since other studies have indicated a similar HIV prevalence among healthcare workers as the general population [35,36] although not age/sex standardised, we do not expect that HIV has confounded the standardised incidence ratio or the absence of association with infection control measures.

### Implications

Effective infection control measures are essential at all health facilities especially in high TB/HIV prevalence settings. These measures should be monitored by validated tools tested locally. In our study, the infection control audit tool did not perform well as a measure of nosocomial transmission risk and poor infection control measures were not associated with TB in healthcare workers. Other strategies to document and monitor TB in healthcare workers should be explored, for instance repeated surveys of TB in healthcare workers which could give an indication of how infection control measures are functioning and/or improving. If the TB burden in healthcare workers in comparison to the general population continues to rise, one would assume that the nosocomial transmission risk is increasing. Such surveys would be in addition to what is already occurring at facilities, requiring additional resources and planning in advance. A national TB prevalence survey would not give an adequate baseline estimate of TB in healthcare workers since too few healthcare workers would be included in such a survey. We thus recommend that the Department of Health should implement a confidential surveillance system for the routine documentation of TB in healthcare workers at facilities. Resources should be made available for diagnostic and therapeutic care for healthcare workers without subjecting them to stigmatisation. Such a routine surveillance system could be used to calculate TB incidence in healthcare workers and monitor trends.
